# Nicotinamide N-methyltransferase (NNMT): a novel therapeutic target for metabolic syndrome

**DOI:** 10.3389/fphar.2024.1410479

**Published:** 2024-06-11

**Authors:** Wei-Dong Sun, Xiao-Juan Zhu, Jing-Jing Li, Ya-Zhong Mei, Wen-Song Li, Jiang-Hua Li

**Affiliations:** Key Lab of Aquatic Training Monitoring and Intervention of General Administration of Sport of China, Physical Education College, Jiangxi Normal University, Nanchang, China

**Keywords:** nicotinamide N-methyltransferase (NNMT), metabolic syndrome (MetS), nicotinamide adenine dinucleotide (NAD^+^), homocysteine (Hcy), obesity, diabetes, hyperlipidemia, hypertension

## Abstract

Metabolic syndrome (MetS) represents a constellation of metabolic abnormalities, typified by obesity, hypertension, hyperglycemia, and hyperlipidemia. It stems from intricate dysregulations in metabolic pathways governing energy and substrate metabolism. While comprehending the precise etiological mechanisms of MetS remains challenging, evidence underscores the pivotal roles of aberrations in lipid metabolism and insulin resistance (IR) in its pathogenesis. Notably, nicotinamide N-methyltransferase (NNMT) has recently surfaced as a promising therapeutic target for addressing MetS. Single nucleotide variants in the NNMT gene are significantly correlated with disturbances in energy metabolism, obesity, type 2 diabetes (T2D), hyperlipidemia, and hypertension. Elevated NNMT gene expression is notably observed in the liver and white adipose tissue (WAT) of individuals with diabetic mice, obesity, and rats afflicted with MetS. Knockdown of NNMT elicits heightened energy expenditure in adipose and hepatic tissues, mitigates lipid accumulation, and enhances insulin sensitivity. NNMT catalyzes the methylation of nicotinamide (NAM) using S-adenosyl-methionine (SAM) as the donor methyl group, resulting in the formation of S-adenosyl-l-homocysteine (SAH) and methylnicotinamide (MNAM). This enzymatic process results in the depletion of NAM, a precursor of nicotinamide adenine dinucleotide (NAD^+^), and the generation of SAH, a precursor of homocysteine (Hcy). Consequently, this cascade leads to reduced NAD^+^ levels and elevated Hcy levels, implicating NNMT in the pathogenesis of MetS. Moreover, experimental studies employing RNA interference (RNAi) strategies and small molecule inhibitors targeting NNMT have underscored its potential as a therapeutic target for preventing or treating MetS-related diseases. Nonetheless, the precise mechanistic underpinnings remain elusive, and as of yet, clinical trials focusing on NNMT have not been documented. Therefore, further investigations are warranted to elucidate the intricate roles of NNMT in MetS and to develop targeted therapeutic interventions.

## 1 Introduction

Metabolic syndrome (MetS) constitutes a multifaceted array of metabolic disorders, manifesting as disturbances in the body’s handling of energy substrates, including proteins, fats, and carbohydrates. As per the latest diagnostic criteria, MetS necessitates the presence of any three out of the following five conditions: elevated fasting glucose or type 2 diabetes (T2D), hypertriglyceridemia, lowered high-density lipoprotein (HDL) cholesterol levels, central obesity, or hypertension (Alberti et al., 2009). Furthermore, these conditions can also result in the prothrombotic state, proinflammatory state, nonalcoholic fatty liver disease, and reproductive disorders (Cornier et al., 2008). It is evident that MetS embodies not a singular malady but rather a confluence of ailments typified by obesity, hypertension, diabetes, and hyperlipidemia (Kane and Sinclair, 2018; Tilg et al., 2020).

The etiology and pathogenesis of MetS remain incompletely elucidated. However, obesity and insulin resistance (IR) are presently acknowledged as significant causative factors in MetS. Nicotinamide N-methyltransferase (NNMT) is a cytoplasmic enzyme that catalyzes the methylation of nicotinamide (NAM) with the methyl donor S-adenosyl-methionine (SAM) to form methylnicotinamide (MNAM) and S-adenosyl-l-homocysteine (SAH) (Alston and Abeles, 1988; Van Haren et al., 2016). NNMT predominantly localizes in adipose tissue and liver (Aksoy et al., 1994). Previous investigations have highlighted elevated NNMT expression in the liver and white adipose tissue (WAT) of diabetic mice and obese, with evidence suggesting that downregulating NNMT expression can mitigate diet-induced IR and obesity (Kraus et al., 2014; Pissios, 2017). Our prior research identified a positive correlation between body mass index (BMI) and urinary MNAM levels (Li and Wang, 2011). Additionally, significant associations were observed between single nucleotide variants in the NNMT gene and energy metabolism, obesity, T2D, hyperlipidemia, and hypertension (Zhu et al., 2016; Zhou et al., 2017; Li et al., 2018; Guan et al., 2021; Liu et al., 2021). Giuliante et al. (Giuliante et al., 2015) reported pronounced elevation of NNMT protein expression, gene expression, and enzyme activity in the adipose tissue of rats afflicted with MetS. Collectively, These results suggest a central position of NNMT in the development of MetS. This review delineates potential mechanisms underlying the NNMT-MetS association and provides insights into the progress of NNMT research within the domain of MetS-related diseases.

## 2 Potential mechanisms linking NNMT to MetS

**FIGURE 1:**
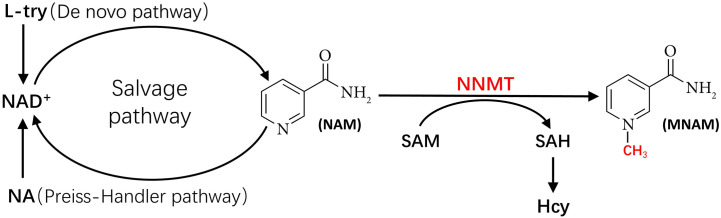
The metabolic pathways linking NNMT to NAD^+^ and to Hcy. Hcy, homocysteine; L-try, L-Tryptophan; MNAM, methylnicotinamide; NA, nicotinic acid; NAD^+^, nicotinamide adenine dinucleotide; NAM, nicotinamide; NNMT, nicotinamide N-methyl transferase; SAH, S-adenosyl-l-homocysteine; SAM, S-adenosyl-methionine.

As shown in Figure 1, NNMT utilizes SAM as a methyl donor to methylate NAM, yielding SAH and MNAM. This enzymatic process has the potential to deplete NAM, a precursor for nicotinamide adenine dinucleotide (NAD^+^), while generating SAH, a precursor for Hcy. Consequently, this reaction could lead to a decline in NAD^+^ levels and an elevation in Hcy levels. Numerous studies have substantiated the significance of reduced NAD^+^ levels and heightened Hcy levels in the pathogenesis of MetS. In subsequent sections, we will delve into the mechanisms through which NNMT activity contributes to diminished NAD^+^ levels and augmented Hcy levels, respectively, and their implications in the context of MetS.

### 2.1 Decreased NAD^+^ levels and MetS

NAD^+^ serves as a critical coenzyme in the conversion of carbohydrates into lipids and fuel oxidation (Belenky et al., 2007). The reduction of NAD^+^ levels potentially represents a pivotal mechanism underlying NNMT’s implication in MetS development. The competitive relationship between NAM methylation and NAD^+^ recycling suggests that NNMT could impede energy expenditure while promoting lipid accumulation. This is attributed to the heightened levels of NNMT potentially rendering NAM irretrievable, thereby constraining NAD^+^-dependent processes (Trammell and Brenner, 2015).

Figure 1 shows the three primary synthetic pathways for NAD^+^: the *de novo* pathway, the Preiss Handler pathway, and the salvage pathway. Among these pathways, the salvage pathway predominates as the principal route for NAD^+^ biosynthesis in mammals (Collins and Chaykin, 1972; Revollo et al., 2007; Mori et al., 2014), contributing to more than 85% of NAD^+^ synthesis (Frederick et al., 2016). Conversely, the *de novo* pathway and Preiss Handler pathway synthesize NAD^+^ using nicotinic acid (NA) and tryptophan as substrates, respectively, albeit in smaller quantities. Notably, NAM serves as the substrate for NAD^+^ synthesis in the salvage pathway (Pissios, 2017). However, NNMT can methylate NAM, converting it to MNAM. Once NAM undergoes methylation by NNMT, it becomes unavailable for NAD^+^ generation via the salvage pathway. Consequently, NNMT activity can directly influence the primary pathway of NAD^+^ production (i.e., the salvage pathway), culminating in diminished NAD^+^ levels.

Studies have consistently reported that inhibiting NNMT activity or downregulating NNMT expression leads to significant increases in NAD^+^ levels in mouse adipocytes (Kraus et al., 2014; Neelakantan et al., 2018) and hepatocytes (Song et al., 2020). In contrast, it has been demonstrated that overexpression of NNMT in mouse liver leads to a decrease in NAD^+^ levels (Komatsu et al., 2018). Regulation of NNMT may affect cancer-related pathways, Huang et al. (Huang et al., 2024) found through *in vitro* experiments that NNMT promotes the migration and invasive ability of esophageal squamous cell carcinoma (ESCC), and NNMT also promotes the process of epithelial mesenchymal transition (EMT) by affecting the post-transcriptional modification of E-calmodulin. Numakura et al. (Numakura and Uozaki, 2024) found that NNMT-positive gastric cancer stromal cells, especially fibroblasts, may promote tumor growth and progression by altering their surrounding epigenetic environment. In human colon cancer cells, silencing NNMT resulted in approximately a 30% increase in NAD^+^ levels (Xie et al., 2014), whereas NNMT overexpression decreased NAD^+^ levels by 30% (Xie et al., 2014). Moreover, Eckert *et al.* (Eckert et al., 2019) reported that the suppression of NNMT resulted in increased NAD^+^ levels in cancer-associated fibroblasts (CAFs). In a study of a mouse unilateral ureteral obstruction (UUO) model, Takahashi *et al.* (Takahashi et al., 2022) observed an elevation in renal NNMT expression and a reduction in NAD^+^ levels. Additionally, Sun et al. (Sun et al., 2022) illustrated that NNMT knockdown in a low-glucose environment enhanced the NAD^+^/NADH ratio in U251 glioma cells.

#### 2.1.1 Association between decreased levels of NAD^+^ and MetS

NAD^+^ functions as a crucial coenzyme in cellular energy metabolism. Loss of NAD^+^ levels results in impairment of mitochondria function, which leads to failure of essential metabolic processes (Klimova and Kristian, 2019; Guimera et al., 2022). This may lead to a less efficient oxidative phosphorylation process, resulting in the production of more free radicals and reactive oxygen species (ROS) (Reid et al., 2018). ROS are a hallmark of oxidative stress and they can damage intracellular proteins, lipids, and DNA, leading to impairment of cellular function and structure, which has been implicated in the development of a variety of diseases including aging, diabetes mellitus, vascular endothelial cell injury, neurodegenerative diseases, and the onset of cellular senescence, strategies to replenish depleted NAD^+^ pools can offer significant improvements of pathologic states (Klimova and Kristian, 2019; Maiese, 2023). A decrease in NAD^+^ levels directly affects the metabolism of the body’s three major energy substances: sugar, fat, and protein, inducing MetS (Covarrubias et al., 2021). Numerous studies have documented a reduction in NAD^+^ levels across various mouse tissues, including adipose (Yoshino et al., 2011), skeletal muscle (Frederick et al., 2015), liver (Trammell et al., 2016), and hypothalamus (Sasaki et al., 2014), concomitant with the development of obesity. Nagahisa *et al.* (Nagahisa et al., 2022) observed that impaired NAD^+^ biosynthesis correlates with the emergence of obesity-associated postprandial hyperglycemia. Specifically, diet-induced obese (DIO) mice exhibited diminished NAD^+^ levels and impaired postprandial glucose metabolism compared to their lean counterparts. Conversely, pharmacological interventions aimed at augmenting NAD^+^ biosynthesis have demonstrated significant improvements in IR and glucose homeostasis (Stromsdorfer et al., 2016; Poddar et al., 2019). Bruckbauer et al*.* (Bruckbauer et al., 2017) investigated the effects of NAM (a precursor of NAD^+^) administration on lipid metabolism in cells and mice. They found that NAM supplementation elevated NAD^+^ levels, thereby ameliorating lipid metabolism and hyperlipidemia. Similarly, Mills *et al.* (Mills et al., 2016) found nicotinamide mononucleotide (NMN) supplemented in mice decreased body weight and increased insulin sensitivity as well as improved plasma lipid profile. Furthermore, Qiu *et al.* (Qiu et al., 2023) reported low levels of NAD^+^ in the aorta from hypertensive patients showing that supplementation with NAD^+^ precursors effectively lowered blood pressure and improved impaired vascular function in both hypertensive patients and mice.

#### 2.1.2 Mechanisms linking decreased NAD^+^ levels to MetS

Decreased NAD^+^ levels are associated with obesity, with studies demonstrating reduced NAD^+^ levels in obese individuals. Elevating NAD^+^ levels can increase lipolysis and reduce body weight. Picard et al. (Picard et al., 2004) observed that overweight and obese patients could activate Sirtuin 1 (SIRT1) and Peroxisome proliferator-activated receptors (PPAR) through NAD^+^ supplementation, resulting in weakened adipogenesis. Barbagallo et al*.* (Barbagallo et al., 2022) demonstrated that NAD^+^ supplementation activates Sirtuin 2 (SIRT2), enhancing the reciprocal inhibition of forkhead box O1 (FOXO1) and PPAR-γ to inhibit adipocyte differentiation and induce fat loss. Uddin et al*.* (Uddin et al., 2017) reported that NAD^+^ supplementation upregulates mitochondrial function, enhancing metabolism and promoting weight loss. Yamaguchi et al*.* (Yamaguchi et al., 2019) created the ANKO (adipocyte-specific nicotinamide phosphoribosyl transferase (Nampt) knockout) mice to elucidate the role of NAD^+^ in the regulation of adaptive thermogenesis and lipolysis. NAMPT serves as the rate-limiting enzyme in NAD^+^ biosynthesis. The study found that ANKO mice, which lack NAMPT in both brown adipose tissue (BAT) and WAT, showed disturbed gene programs involving thermogenesis and mitochondrial function in BAT, resulting in a dampened thermal response. Furthermore, the absence of NAMPT in WAT resulted in a significant reduction in adrenergic-induced lipolysis. These results underscore the critical role of adipose NAD^+^ in modulating adaptive thermogenesis, lipolysis, and overall energy metabolism.

Decreased NAD^+^ levels have been associated with hyperglycemia. NAD^+^ plays a pivotal role in regulating the insulin signaling pathway and enhancing mitochondrial function, thereby contributing to the regulation of blood glucose levels. However, a decline in NAD^+^ levels can precipitate a NAD^+^/NADH redox imbalance, resulting in an accumulation of excess NADH. This surplus of electron donors in the mitochondrial electron transport chain can overwhelm and impair mitochondrial complex I (Yan et al., 2016). Furthermore, excessive accumulation of NADH in conjunction with reduced NAD^+^ availability can inhibit glyceraldehyde-3-phosphate dehydrogenase (GAPDH) activity in the glycolytic pathway, leading to glucose shunting to the glycolytic branch pathway (Yan et al., 2016; Song et al., 2019). This diversion activates the polyol pathway while diminishing cytoplasmic NAD^+^ levels (Luo et al., 2015). The hexosamine pathway can be activated to enhance O-GlcNAc acylation, potentially leading to IR (Chen Y. et al., 2019). Additionally, Protein kinase C (PKC) pathway activation can induce insulin receptor substrate 1 (IRS-1) phosphorylation, inhibiting insulin signaling through downstream effectors such as Akt, phosphoinositide 3-kinase (PI3K), and glucose transporter 4 (GLUT-4) (Ritter et al., 2015; Kolczynska et al., 2020). Furthermore, the formation of advanced glycosylation end products can exacerbate IR, pancreatic β-cell dysfunction, and cellular apoptosis (Nowotny et al., 2015).

Decreased levels of NAD^+^ have been implicated in hypertension. NAD^+^ exhibits vasorelaxant effects and diminishes oxidative stress, potentially contributing to the regulation of blood pressure. It is imperative to note that while these effects suggest a potential benefit, further research is needed to clarify the relationship between NAD^+^ and hypertension. Investigations conducted on rat thoracic aorta and porcine coronary arteries have demonstrated that elevated levels of NAD^+^ induce concentration-dependent vasorelaxation in endothelial cells (ECs) across various arterial beds. This vasorelaxation is mediated through actions on purinergic receptors, specifically adenosine receptors (Alefishat et al., 2015). Moreover, NAD^+^ regulates the activity of NADPH oxidase, a significant ROS source in vascular cells, thereby playing a key role in the modulation of vascular tone (Reid et al., 2018). Elevated oxidative stress fosters the proliferation and hypertrophy of vascular smooth muscle cells, as well as collagen deposition, culminating in vascular medial thickening and constriction. Enhanced oxidative stress may also impair endothelial function, leading to diminished endothelium-dependent vasorelaxation and heightened vascular contractility. These observations underscore the potential link between heightened oxidative stress and the pathogenesis of hypertension (Grossman, 2008). Consequently, by mitigating oxidative stress and enhancing vascular function, NAD^+^ holds promise as a therapeutic target for blood pressure regulation.

Decreased levels of NAD^+^ have been associated with dyslipidemia. While direct evidence of NAD^+^’s impact on lipid levels is limited, several studies have indicated that supplementation with NAD^+^ precursors can ameliorate lipid profiles. Meta-analyses have shown that supplementation with NAD^+^ precursors, including NMN, nicotinamide riboside (NR), NA, and NAM, significantly reduces levels of triglycerides (TG), total cholesterol (TC), and low-density lipoprotein (LDL), while concurrently increasing high-density lipoprotein levels (Zhong et al., 2022). Imi et al (Imi et al., 2023). Demonstrated that administration of NMN, an NAD^+^ precursor, markedly reduced subcutaneous fat mass in mice fed a high-fat diet (HFD). The study showed a decrease in the size of adipocytes in subcutaneous adipose tissue after NMN treatment. Furthermore, the treatment with NMN resulted in a significant upregulation of adipose triglyceride lipase (ATGL) expression in subcutaneous fat, which is in line with the observed alterations in fat mass and adipocyte size (Imi et al., 2023). These findings suggest that NAD^+^ may exert beneficial effects on lipid profiles by modulating the expression of lipid-metabolizing enzymes.

### 2.2 Increased Hcy levels and MetS

Hyperhomocysteinemia (HHcy) stands as a well-established independent risk factor for cardiovascular and cerebrovascular diseases (Dubchenko et al., 2019). Hence, understanding the potential pathophysiological interplay between Hcy levels and MetS is of paramount’ importance. Moreover, there exists evidence linking elevated Hcy levels to an increased risk of MetS (Sreckovic et al., 2017). Esteghamati et al*.* (Esteghamati et al., 2014) documented a significantly relationship among elevated Hcy levels and major manifestations of MetS, including impaired glucose tolerance (IGT), abdominal obesity, elevated blood pressure, and hypertriglyceridemia. Consequently, the elevation in Hcy levels induced during NAM methylation emerges as another crucial mechanism underlying the involvement of NNMT in MetS development. Figure 1 illustrates NNMT utilizing SAM as a methyl donor to methylate NAM, yielding MNAM and SAH. Subsequently, SAH is converted to Hcy by SAH hydrolase (SAHase) (Aksoy et al., 1994; Okamoto et al., 2003; Souto et al., 2005). Therefore, heightened NNMT activity leads to elevated blood Hcy levels. A genome-wide scanning study conducted by Souto et al*.* (Souto et al., 2005) on Spanish families corroborated NNMT as the principal genetic determinant of plasma Hcy levels.

#### 2.2.1 Association between increased Hcy levels and MetS

While the precise mechanism underlying how HHcy contributes to MetS remains incompletely understood, substantial evidence supports HHcy as a major risk factor for MetS development. Lee et al*.* (Lee et al., 2021) conducted comprehensive analyses utilizing datasets such as the Korean Association Resource (KARE), Health Examinees (HEXA), and Cardiovascular Disease Association Study (CAVAS), employing both one-sample Mendelian Randomization (MR) and two-sample MR methods. Their findings consistently demonstrated a significant association between HHcy and increased risk of MetS. Similarly, Ulloque-Badaracco et al*.* (Ulloque-Badaracco et al., 2023) identified a significant correlation between elevated Hcy levels and MetS. You et al*.* (You et al., 2023) observed a substantial correlation between HHcy and MetS prevalence among Chinese older adults. Liu et al*.* (Liu et al., 2022) conducted a longitudinal study involving Chinese community residents aged ≥65 years, revealing that HHcy escalates the risk of MetS, particularly among individuals with abdominal obesity. Piazzolla et al*.* (Piazzolla et al., 2019) conducted a cross-sectional analysis of MetS patients, noting elevated Hcy levels in a considerable proportion of cases. Butkowski et al*.* (Butkowski et al., 2017) investigated the interplay among Hcy, oxidative stress processes, and MetS, elucidating a positive association between Hcy levels and the presence of MetS factors. Yakub et al*.* (Yakub et al., 2014) evaluated a cohort of rural Nepalese children aged 6–8 years, discovering an elevated risk of MetS associated with increased Hcy levels. These collective findings underscore the significant role of HHcy in the pathogenesis of MetS across diverse populations and age groups.

#### 2.2.2 Mechanisms linking increased Hcy levels to MetS

Elevated Hcy levels exert inhibitory effects on the insulin signaling pathway, primarily by inducing IR through the cysteine-homocysteinylation of the pro-insulin receptor. Hcy interacts with cysteine-825 residues situated on the pro-insulin receptor within the endoplasmic reticulum (ER), thereby disrupting the formation of initial disulfide bonds. This cysteine-homocysteinylation event perturbs the pro-insulin receptor and furin protease interaction within the Golgi apparatus, consequently inhibiting the cleavage of the pro-insulin receptor. Elevated Hcy levels in mice result in diminished levels of mature insulin receptors across various tissues, culminating in IR and the onset of T2D (Zhang et al., 2021).

1Increased Hcy levels promote oxidative stress. Elevated Hcy leads to an increase in the generation of mitochondrial ROS, resulting in oxidative damage at the cellular and molecular levels, and suppression of antioxidant enzyme systems (Kaplan et al., 2020). Hcy triggers oxidative stress by multiple means, such as generating ROS directly through auto-oxidation with transition metals, activating oxidative pathways, and blocking antioxidant pathways (Perna et al., 2003; Esse et al., 2019; Ostrakhovitch and Tabibzadeh, 2019). This leads to damage to tissue cells, including vascular ECs (Incalza et al., 2018), adipocytes (Furukawa et al., 2004), and pancreatic β-cells (Gerber and Rutter, 2017). Ultimately, this results in the development of MetS.

Elevated Hcy levels have been implicated in perturbing fat metabolism, presenting a potential link between Hcy and adipose tissue dysfunction, which further contributes to cardiovascular disease risk. Recent research indicates that Hcy disrupts the breakdown of fats in fat cells by activation of the AMP-activated protein kinase (AMPK) pathway (Wang et al., 2011). Exposure to Hcy leads to a decrease in glycerol and free fatty acid (FFA) release in fully differentiating 3T3-L1 and primary adipose cells in a dose-dependent manner. Notably, the inhibitory effects of Hcy on lipolysis are mediated through AMPK activation in adipocytes, underscoring the significance of AMPK in mitigating the impacts of Hcy on fat metabolism (Wang et al., 2011). Furthermore, elevated plasma Hcy levels have been associated with decreased levels of HDL-cholesterol and disturbances in plasma lipid profiles, potentially leading to fatty liver accumulation (Obeid and Herrmann, 2009). Hypomethylation induced by HHcy appears to contribute to lipid deposition in tissues. Investigating mechanisms underlying dysregulated lipid metabolism, Visram et al*.* (Visram et al., 2018) established a model of HHcy in yeast and showed that Hcy supplementation resulted in increased cellular levels of fatty acids and triacylglycerols. Furthermore, the model demonstrated increased resistance to cerulenin, a fatty acid synthase inhibitor, and lower levels of condensing enzymes linked to the synthesis of very long-chain fatty acids (Visram et al., 2018). These findings underscore the intricate relationship between Hcy and lipid metabolism dysregulation, shedding light on potential mechanisms underlying adipose tissue dysfunction and its implications for cardiovascular health.

Increased Hcy levels activate inflammatory response. HHcy activates the release of inflammatory factors, induces leukocyte chemotaxis and activation, and causes ER stress. This results in an increased inflammatory response, which damages tissue cells, such as vascular ECs and pancreatic β-cells, ultimately leading to Mets. High levels of Hcy can activate the expression and release of inflammatory factors, leading to vascular endothelial dysfunction (Balint et al., 2020). Numerous *in vitro* studies have corroborated the ability of Hcy to induce the release of inflammatory cytokines from ECs, including interleukin-6, interleukin-8, and tumor necrosis factor-α (Kamat et al., 2013; Han et al., 2015; Li et al., 2016). This inflammatory response within the vasculature may stem from the activation of Nuclear Factor-kappa β (NFkβ) (Zhao et al., 2017). Animal models of HHcy corroborate these findings. For instance, in a murine model, Hofmann *et al.* (Hofmann et al., 2001) reported that HHcy exacerbates vascular inflammation and accelerates atherosclerosis. Additionally, Wu et al*.* (Wu et al., 2019) demonstrated in human umbilical vein ECs and arteries of HHcy mice that Hcy triggers the expression of ER oxidoreductin-1α (Ero1α), leading to ER stress and subsequent inflammation. Notably, Ero1α knockdown has been found to mitigate HHcy-induced ER oxidative stress and inflammation.

## 3 Study progress of NNMT in MetS-related diseases

Metabolic Syndrome (MetS) represents a constellation of metabolic disorders arising from intricate interplays between genetic predisposition and environmental influences. While the precise etiology remains incompletely understood, central obesity and insulin resistance (IR) stand prominently as pivotal contributors to MetS onset. Additionally, dyslipidemia, hypertension, and other factors are recognized as potential drivers of MetS pathogenesis (Kane and Sinclair, 2018; Tilg et al., 2020). The ensuing sections will delineate the advancements in NNMT research pertaining to obesity, diabetes, hyperlipidemia, and hypertension.

### 3.1 NNMT and obesity

Obesity stands as a pathological state characterized by the excessive accumulation of body fat and constitutes a significant contributor to MetS. Emerging evidence suggests a pivotal role of NNMT in the context of obesity. Initial metabolomics investigations unveiled a noteworthy correlation between urinary MNAM levels and BMI (Salek et al., 2007; Li and Wang, 2011). Subsequent studies by Liu *et al.* (Liu et al., 2015) extended these findings, revealing a positive association between serum MNAM levels and obesity. These observations hint at a potential linkage between obesity and augmented NNMT activity, given MNAM’s production during NNMT enzymatic reactions. This notion gains further support from recent experimental endeavors. Multiple studies have reported a conspicuous upregulation of NNMT expression in WAT among mice prone to obesity compared to their obesity-resistant counterparts (Alexander et al., 2006; Svenson et al., 2007; Wu et al., 2009). Notably, Lee et al. (Lee et al., 2005) documented significantly elevated NNMT expression in abdominal subcutaneous adipocytes of obese Pima Indians relative to non-obese counterparts. Furthermore, Kraus et al*.* (Kraus et al., 2014) elucidated that NNMT silencing significantly reduced relative adiposity in mice by approximately 47%. They proposed that heightened NNMT expression in WATs and/or liver could potentially serve as a contributory factor to obesity. Additionally, it has been documented that NNMT knockdown significantly mitigated both body weight and adiposity in female mice subjected to a Western diet, characterized by a composition of 47% kcal of fat and 34% kcal of carbohydrate (Brachs et al., 2019). Moreover, Roberti et al*.* (Roberti et al., 2023) highlighted the preventive effects of NNMT blockade during the initial phases of adipogenesis, which inhibited preadipocyte differentiation into adipocytes in response to glucocorticoids. Furthermore, NNMT gene knockout resulted in a significant reduction in adipogenesis. Dong et al. demonstrated that knockdown of the Nmnat1 gene in hepatic cells leads to depletion of nuclear NAD^+^, thereby causing mitochondrial dysfunction (Dong et al., 2024). NMNAT is an essential key enzyme in the NAD^+^ salvage pathway. Its deficiency results in reduced NAD^+^ synthesis and accumulation of its substrate, NAM, leading to increased NNMT activity and further decline in NAD^+^ levels. NAD^+^ plays a crucial role in maintaining mitochondrial function and energy metabolism. Decreased NAD^+^ levels can impair the efficiency of the mitochondrial respiratory chain, resulting in reduced energy production, which may exacerbate obesity and related metabolic disorders (Amjad et al., 2021). Our previous research investigated the association between NNMT gene sequence variants and obesity, revealing significant findings. Specifically, investigations on the Chinese Han population identified the rs10891644 variant as significantly associated with obesity susceptibility, with heterozygous individuals (GT type) exhibiting heightened vulnerability (Zhou et al., 2017). Analogously, Bañales-Luna *et al* (Bañales-Luna et al., 2020). Reported a significantly correlation of the variant rs694539 of the NNMT gene sequence with BMI values in the Mexican population, with carriers of the GG and AG genotypes having an increased susceptibility to obesity.

**FIGURE 2:**
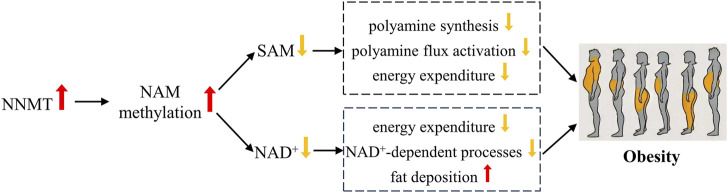
Possible mechanisms of NNMT associated with obesity.

Obesity arises from an imbalance in energy metabolism, wherein energy intake surpasses energy expenditure. As shown in Figure 2, NNMT’s influence on obesity predominantly stems from its regulatory role in energy metabolism, particularly through NAM methylation, which significantly impacts energy modulation. Upon methylation, NAM becomes irretrievable for NAD^+^ synthesis (Trammell and Brenner, 2015). NAD^+^ is a vital coenzyme in fuel metabolism and the transformation of carbohydrate into lipid (Houtkooper et al., 2010). The relationship between NAM metabolism and NAD^+^ utilization indicates that elevated NNMT expression may inhibit energy expenditure and promote fat deposition by limiting NAD^+^-dependent processes (Trammell and Brenner, 2015). Our prior investigations have unveiled substantial correlations between NNMT genetic variants and resting energy expenditure (Zhu et al., 2016), as well as maximal oxygen uptake (Teperino et al., 2010) in the Chinese populace. Bañales-Luna et al. (Bañales-Luna et al., 2020) recently corroborated these findings, discovering a significant link between NNMT genetic variants and resting energy expenditure in Mexican individuals. Additionally, Kraus *et al.* (Kraus et al., 2014) shed light on NNMT’s regulation of energy metabolism and associated mechanisms through laboratory experiments. *In vitro* studies revealed that diminishing NNMT expression via RNA interference (RNAi) and inhibiting NNMT with MNAM substantially augmented oxygen consumption (OC) in adipocytes. Conversely, adipocytes exhibited a considerable decrease in oxygen consumption upon NNMT overexpression (Kraus et al., 2014). In animal models, mice with decreased NNMT levels exhibited heightened energy expenditures compared to control mice of similar weights (Kraus et al., 2014). NNMT modulates energy expenditure by regulating SAM and NAD^+^ during NAM methylation. SAM and NAD^+^ serve as critical cofactors in cellular energy metabolism and redox states (Jell et al., 2007). SAM facilitates polyamine synthesis by providing propylamine and adding a methyl group for histone methylation (Doench et al., 2003). Enhanced polyamine flux activation leads to elevated energy expenditure via polyamine acetylation, utilizing Acetyl-coenzyme A (acetyl-CoA) to generate acetylpolyamines (Jackson et al., 2003). Inhibition of NNMT augments SAM and NAD^+^ levels, resulting in the upregulation of polyamine flux activation and increased energy expenditure (Kraus et al., 2014).

### 3.2 NNMT and diabetes

Diabetes is caused by a fundamental disturbance in the metabolism of glucose and is characterised by IGT, elevated fasting blood glucose, and inadequate insulin secretion or IR (Tripathi and Srivastava, 2006; Goldberg and Mather, 2012). MetS is a multifaceted condition that is defined as IR, IGT, T2D, or impaired fasting glucose (IFG) along with the existence of two or more of the followings: hypertension, microalbuminuria, hyperlipidemia, and obesity (Giuliante et al., 2015). It is evident that disorders of glucose metabolism in the form of diabetes and IR are the core elements of the MetS.

Multiple trials have robustly shown a strong association of NNMT with IR and T2D (Kraus et al., 2014; Kannt et al., 2015; Liu et al., 2015). Our previous study also identified a significant association between the NNMT genetic variant at rs1941404 and T2D in the Chinese Han population (Li et al., 2018). Additionally, NNMT has been shown to play a causal role in the development of T2D through quantitative trait locus mapping in mice (Yaguchi et al., 2005). Elevated levels of MNAM were detected in the urine (Li et al., 2018) and serum (Kannt et al., 2015) of individuals with T2D, suggesting an increase in NNMT activity during T2D development. Further direct evidence supporting this relationship is primarily derived from the reports outlined below. Kannt et al. (Souto et al., 2005) demonstrated an upregulation of NNMT expression in the WAT of individuals with IR or T2D. Additionally, a noteworthy correlation was found among plasma levels of MNAM and both the degree of IR and the expression levels of NNMT in WAT. Kraus *et al.* (Kraus et al., 2014) reported an increase in NNMT expression levels in the livers and WATs of T2D mice. They also found that knockdown of NNMT improved insulin sensitivity and glucose tolerance in the T2D mice. The role of NNMT in the development of T2D was further confirmed by Hong et al*.* (Hong et al., 2015). Their study revealed that hepatocyte glucose output decreased by 50% with NNMT knockdown, while NNMT overexpression increased it by 1.4-fold. Meanwhile, they observed a significantly lower overnight fasted glucose level in the livers of C57BL6/J mice with NNMT knockdown (Hong et al., 2015).

The potential mechanisms underlying NNMT’s regulation of glucose metabolism are primarily derived from two key reports. Kraus *et al.* (Kraus et al., 2014) demonstrated that reduced NNMT expression enhances glucose tolerance and improves insulin sensitivity in DIO mice. The role of NNMT in gluconeogenesis was further elucidated by Hong et al*.* (Hong et al., 2015). Their results revealed that decreasing NNMT expression in primary hepatocytes led to a reduction in the expression of phosphoenolpyruvate carboxykinase 1 (Pck1) and glucose-6-phosphatase (G6pc), resulting in decreased glucose output. Conversely, increasing NNMT expression in primary hepatocytes resulted in elevated glucose output and increased expression of Pck1 and G6pc. Furthermore, Hong *et al.* (Hong et al., 2015) observed that mice subjected to NNMT knockdown exhibited reduced fasting glucose levels and diminished conversion of pyruvate to glucose. Such observations indicate that NNMT plays a beneficial role in regulating gluconeogenesis in hepatocytes.

**FIGURE 3:**
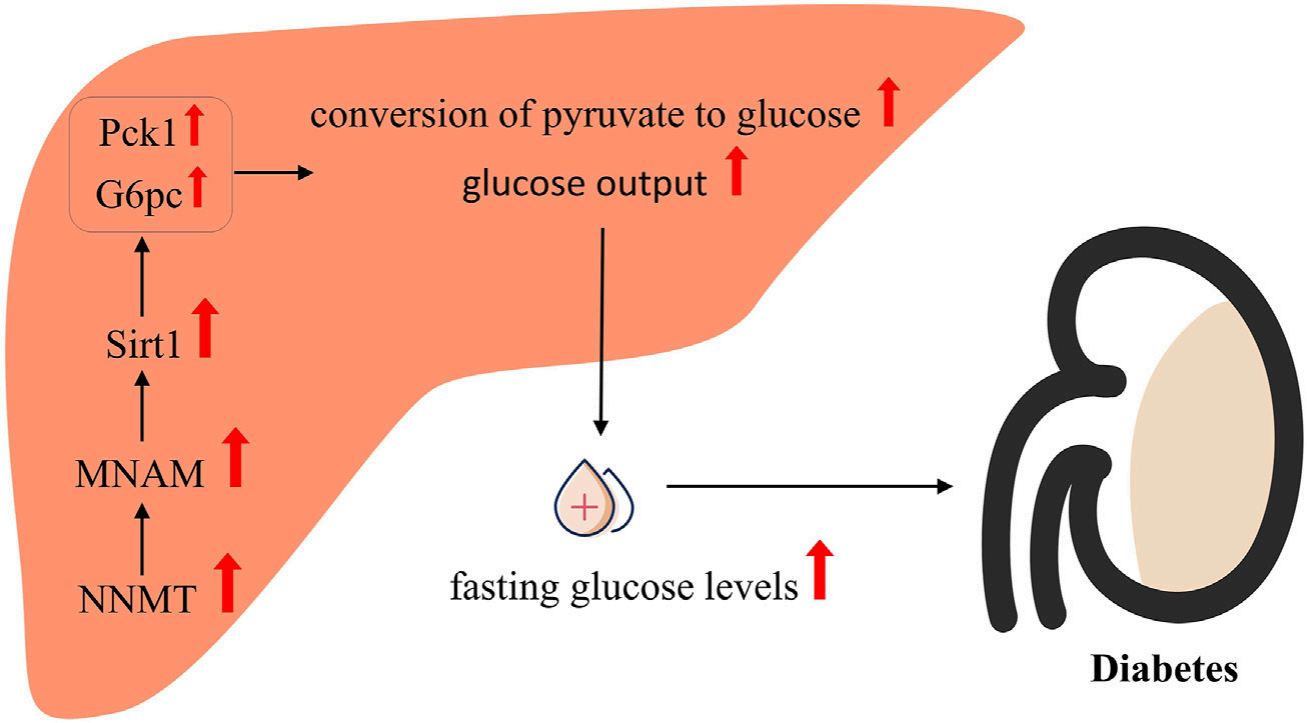
Possible mechanisms of NNMT associated with diabetes.

As shown in Figure 3, in elucidating the regulatory mechanism of NNMT on glucose metabolism, Hong *et al.* (Hong et al., 2015) proposed that NNMT’s product, MNAM, mediates its regulatory impact, with Sirt1 playing a pivotal role in this process. Their findings revealed a significant reduction in glucose production and expression of both G6pc and Pck1 in hepatocytes with NNMT overexpression and concurrent Sirt1 inhibition, compared to hepatocytes with NNMT overexpression alone. Furthermore, the downregulation of G6pc and Pck1 expressed by NNMT knockdown was reversed by the upregulation of Sirt1. These observations underscore the essential role of Sirt1 in mediating NNMT’s regulatory effect on glucose metabolism. Additionally, a significant correlation between Sirt1 protein expression and NNMT expression was noted in hepatocytes. *In vitro* experiments demonstrated that NNMT overexpression significantly increased Sirt1 protein expression in primary hepatocytes, while NNMT knockdown led to a notable decrease in Sirt1 protein expression. Consistent with these findings, *in vivo* experiments revealed a significant reduction in Sirt1 protein expression in the livers of mice upon NNMT knockdown. Furthermore, MNAM, a product of NNMT, was found to playing a key part in this process. Similar to the effects of NNMT overexpression, hepatocytes treated with MNAM exhibited significant increases in glucose production and expression of Sirt1, G6pc, and Pck1. Notably, the alterations induced by MNAM treatment were abrogated upon Sirt1 knockdown. These results collectively indicate that Sirt1 is indispensable for NNMT-mediated regulation of glucose metabolism, and both NNMT and MNAM have the ability to upregulate Sirt1 expression.

### 3.3 NNMT and hyperlipidemia

Hyperlipidemia is a medical condition characterized by elevated levels of one or more plasma lipids, including TG, cholesterol, cholesteryl esters, phospholipids, and LDL, coupled with a reduction in HDL levels (P et al., 2021). It represents one of the complications associated with MetS. Despite limited research on the association between NNMT and hyperlipidemia, compelling evidence suggests its potential role in lipid metabolism. Notably, studies have shown that knocking down NNMT in adipose tissues and livers of mice fed a high-fat diet resulted in decreased serum TG and FFA levels (Kraus et al., 2014). Furthermore, our previous analysis of 19 tagSNPs in the NNMT gene sequences of Chinese Han patients revealed a significant association between the variant at rs1941404 and hyperlipidemia. Specifically, individuals carrying the CC genotype at this locus were identified as the susceptible population. The regulatory effect of the rs1941404 variant on resting energy expenditure offers a plausible explanation for the observed association between NNMT and hyperlipidemia. These findings underscore the potential involvement of NNMT in lipid metabolism regulation and its relevance to the development of hyperlipidemia, a key component of MetS (Zhu et al., 2016).

**FIGURE 4:**
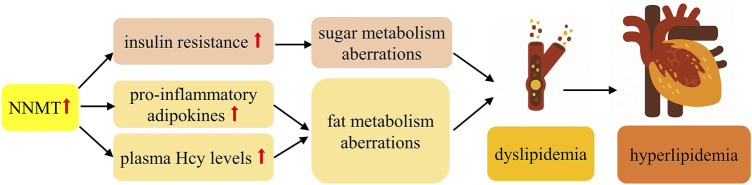
Possible Mechanisms of NNMT associated with hyperlipidemia.

The etiology of hyperlipidemia remains incompletely elucidated; however, it is widely believed to be linked to aberrations in both sugar and fat metabolism. Epidemiological investigations have underscored a significant correlation between hyperlipidemia and conditions such as obesity or disruptions in energy metabolism (Zhu et al., 2016). Numerous studies have underscored a robust association between obesity and dyslipidemia (Vekic et al., 2019). As shown in Figure 4, Evidence suggests that dyslipidemia predominantly stems from IR and the influence of pro-inflammatory adipokines (Klop et al., 2013). Consequently, it is plausible that NNMT’s involvement in hyperlipidemia is mediated through its impact on both sugar and fat metabolism. Moreover, NNMT might contribute to hyperlipidemia by influencing plasma Hcy levels. Multiple studies have highlighted a pronounced link between plasma Hcy levels and hyperlipidemia (Sreckovic et al., 2017; Hasan et al., 2019). Additionally, Mondal *et al.* (Mondal et al., 2018) demonstrated that elevated homocysteine levels can directly modulate lipid metabolism and induce hyperlipidemia in rats through Hcy administration (50 mg/kg/d).

### 3.4 NNMT and hypertension

Hypertension is another common complication of MetS. Although there are limited reports regarding the association between NNMT and hypertension, our research has identified a significant association between a SNP in the NNMT gene (rs1941404) and hypertension within the Chinese Han population (Guan et al., 2021).

**FIGURE 5:**
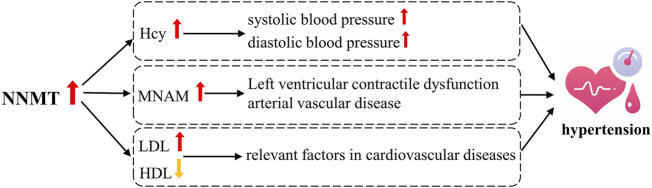
Possible Mechanisms of NNMT associated with hypertension.

While direct evidence linking NNMT activity to hypertension is currently lacking, numerous studies have established a strong association between NNMT activity and various metabolic disorders, including cardiovascular diseases (Li et al., 2006; Williams et al., 2012). For instance, Liu et al. (Liu et al., 2017) demonstrated a significant association between serum MNAM levels and coronary artery disease, as well as left ventricular systolic dysfunction in Chinese patients (Liu M. et al., 2018). Additionally, Bubenek *et al.* (Bubenek et al., 2012) reported a robust association between NNMT expression, serum MNAM levels, and the onset and progression of peripheral arterial occlusive disease. Notably, NNMT expression has been found to be positively correlated with LDL levels and negatively correlated with HDL levels, both of which are relevant factors in cardiovascular diseases (Herfindal et al., 2020; Kornev et al., 2023; Rus et al., 2023). As shown in Figure 5, left ventricular contractile dysfunction and arterial vascular disease are significant contributors to the development of hypertension, suggesting a potential pathway through which NNMT may influence hypertension. Moreover, elevated plasma Hcy levels have been significantly associated with hypertension (Van Guldener et al., 2003), particularly with systolic blood pressure (SBP) (Esteghamati et al., 2014). Junedi et al. (Junedi et al., 2022) reported higher Hcy levels in hypertensive cases compared to controls. Moreover, increased plasma Hcy levels were associated with elevated SBP, diastolic blood pressure (DBP), and a higher prevalence of hypertension among middle-aged and elderly individuals of Chinese descen (Hidru et al., 2019). Notably, patients with HHcy exhibit significantly higher blood pressure compared to those without HHcy, and serum Hcy levels have been positively correlated with blood pressure in Wistar rats (Shi et al., 2019). Thus, influencing Hcy levels may represent another important pathway through which NNMT is implicated in the development of hypertension.

## 4 NNMT as a therapeutic target for MetS

### 4.1 RNAi drugs

As previously stated, NNMT activity results in reduced NAD^+^ production and increased Hcy production. Studies have shown that knocking down the NNMT gene leads to increased expenditure of energy in adipocytes (Kraus et al., 2014) and decreased output of glucose from hepatocytes (Hong et al., 2015). Additionally, in animal models, NNMT gene knockdown has been found to improve insulin sensitivity (IS), glucose tolerance (GT), and overnight fasting glucose (OFG) levels, while also reducing lipid accumulation and triglyceride content (Kraus et al., 2014; Hong et al., 2015; Xu et al., 2022). These findings suggest that NNMT may represent a promising therapeutic target for the development of RNAi drugs aimed at preventing or treating MetS (Iyamu and Huang, 2020; Van Haren et al., 2021; Harikrishna and Venkitasamy, 2023). Some studies have used endogenous small interfering RNAs (miRNAs) to inhibit NNMT and competitive endogenous RNAs (ceRNAs) as “sponges” of miRNAs to reduce the inhibitory effect of miRNAs on target genes. MiRNAs are small noncoding RNAs that regulate gene expression via recognition of cognate sequences and interference of transcriptional, translational or epigenetic processes (Chen L. et al., 2019), miRNAs control mRNA expression, miRNAs are also capable of targeting non-coding RNAs, including long non-coding RNAs and miRNAs (Hill and Tran, 2021). Recently Yu et al. (Yu et al., 2021) and Wang et al. (Wang et al., 2021) identified miR-29b-3p and miR-378g as potential miRNAs upstream of NNMT by bioinformatics analysis. It was confirmed by dual luciferase reporter assay that miR-29b-3p and miR-378g directly target the 3′UTR of NNMT and negatively regulate NNMT expression. In osteoporosis, both miR-29b-3p and miR-378g regulate osteogenic differentiation of BMSCs by targeting NNMT. Inhibition of miR-29b-3p or miR-378g upregulates NNMT expression and promotes osteogenic differentiation of bone marrow mesenchymal stem cells (BMSCs), whereas overexpression of miR-29b-3p or miR-378g downregulates NNMT expression and inhibits osteogenic differentiation of BMSCs. Competitive endogenous RNAs (ceRNAs) act as “sponges” for miRNAs, ceRNAs can bind to miRNAs and reduce the abundance of miRNAs in the cell, thus reducing the inhibitory effect of miRNAs on target genes. Yu et al. (Yu et al., 2021) and Wang et al. (Wang et al., 2021) found that XIST and HOTAIR are long non-coding RNAs (lncRNAs) with significantly elevated expression in the serum of patients with osteoporosis, and that XIST and HOTAIR acted as ceRNAs (competing endogenous RNAs) to upregulate NNMT expression by adsorbing miR-29b-respectively. 3p and miR-378g, respectively, to upregulate the expression of NNMT, thereby inhibiting the osteogenic differentiation of BMSCs.

However, RNAi therapeutics still face technical obstacles, such as achieving efficient delivery of RNAi drugs targeting organs or tissues and overcoming the off-target interactions (Doench et al., 2003; Jackson et al., 2003; Persengiev et al., 2004; Scacheri et al., 2004), as well as addressing the issues of sequence-dependent and chemical-dependent toxicity (Grimm et al., 2006; Jackson et al., 2006). While certain RNAi drugs aimed at NNMT have shown efficacy *in vitro* and animal studies, there are no reports of such products being utilized in clinical trials thus far. It is worth noting, however, that the Food and Drug Administration (FDA) of the U.S. has approved over 10 RNAi drugs (Roberts et al., 2020). The technical barriers to RNAi therapies are gradually being resolved, implying that developing RNAi drugs that target NNMT remains a viable approach to prevent or treat MetS.

### 4.2 Small molecule inhibitors targeting NNMT

**FIGURE 6:**
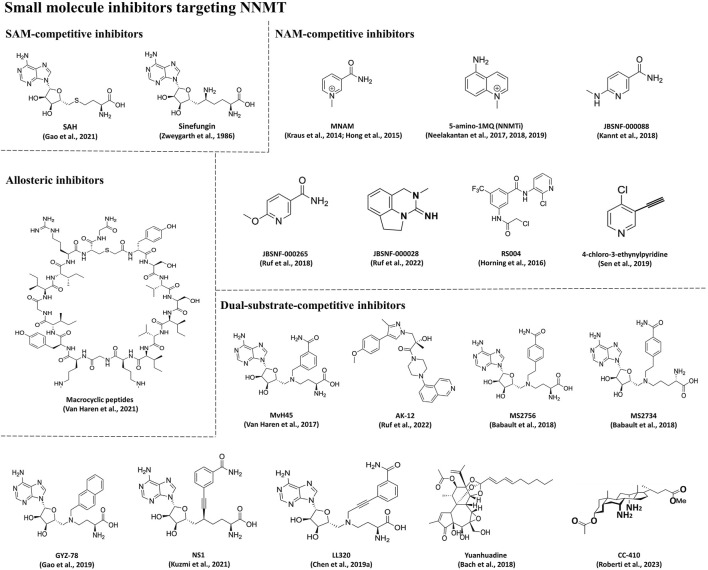
Chemical structures of small molecule inhibitors targeting NNMT.

In addition to RNAi drugs, the development of small molecule inhibitors targeting NNMT represents a current research focus for the treatment of MetS-related diseases. As shown in Table 1 and Figure 6, the small molecule inhibitors targeting NNMT can be categorized into SAM-competitive inhibitors, NAM-competitive inhibitors, Dual-substrate-competitive inhibitors and isomerization inhibitors.

**TABLE 1:** Overview of the small molecule inhibitors targeting NNMT.

NNMT inhibitor type	Compound
SAM-competitive inhibitors	SAH (Gao et al., 2021), sinefungin^a^ (Zweygarth et al., 1986)
NAM-competitive inhibitors	MNAM (Kraus et al., 2014; Hong et al., 2015), 5-amino-1MQ (NNMTi) (Neelakantan et al., 2017; Neelakantan et al., 2018; Neelakantan et al., 2019), **JBSNF-000088** (Kannt et al., 2018), **JBSNF-000265** (Ruf et al., 2018), **JBSNF-000028** (Ruf et al., 2022), **RS004** (Horning et al., 2016), 4-chloro-3-ethynylpyridine (Sen et al., 2019)
Dual-substrate-competitive inhibitors	MvH45 (Van Haren et al., 2017), **AK-12** (Ruf et al., 2022), **MS2756** (Babault et al., 2018), **MS2734** (Babault et al., 2018), **GYZ-78** (Gao et al., 2019), **NS1** (Kuzmi et al., 2021), **LL320** (Chen et al., 2019a), Yuanhuadine^a^ (Bach et al., 2018), **CC-410** (Roberti et al., 2023)
Allosteric inhibitors	Macrocyclic peptides (Van Haren et al., 2021)

^a^ A natural inhibitor.

#### 4.2.1 SAM-competitive inhibitors

SAM-competitive inhibitors compete with the substrate SAM for the binding site in NNMT. There are two main types of such inhibitors, SAH (Gao et al., 2021) and sinefungin (Zweygarth et al., 1986) (Table 1; Figure 6), but they are non-selective and inhibit all methyltransferases (Van Haren et al., 2016). However, SAH demonstrates activity solely in enzyme-based biochemical assays; its activity is diminished in cellular assays owing to its swift degradation to adenosine and Hcy by SAH hydrolase (Gao et al., 2021). Sinefungin, a naturally occurring compound derived from *Streptomyces*, serves as a SAM-mimicking methyltransferase inhibitor. However, it exhibits low cell membrane permeability and demonstrates substantial toxicity in animal models, thereby constraining its potential application as a therapeutic agent (Zweygarth et al., 1986).

#### 4.2.2 NAM-competitive inhibitors

NAM-competitive inhibitors are substances that can compete with the substrate NAM for the binding site in NNMT. Table 1 and Figure 6 lists several inhibitors in this category, but the most frequently reported ones are MNAM and 5-amino-1MQ (NNMTi).

MNAM, a natural inhibitor of NNMT, is generated through the enzymatic reaction catalyzed by NNMT (Gao et al., 2021). In a study conducted by Swaminathan et al. (Swaminathan et al., 2017), the X-ray crystal structure of a ternary complex of NNMT bound with MNAM was elucidated, revealing that MNAM binds to the active site of NNMT, thereby impeding NAM binding and suppressing NNMT’s enzymatic activity. MNAM has been shown *in vitro* to be a significant enhancer of energy expenditure as well as a regulator of glucose metabolism. Kraus *et al.* (Kraus et al., 2014) used MNAM to inhibit NNMT activity in adipocytes and found that it induced an increase in oxygen consumption. However, its effectiveness inside the body is controversial. Hong *et al.* (Hong et al., 2015) demonstrated that MNAM administration prevents changes in fasting blood glucose levels and insulin sensitivity in mice fed a HFD. Moreover, MNAM inhibits the biosynthesis of fatty acids and cholesterol, along with suppressing the expression of lipogenic and cholesterol-synthesizing genes, leading to significant reductions in liver cholesterol and TC levels. However, Przyborowski et al*.* (Przyborowski et al., 2015) reported that administering MNAM (100 mg/kg) to diabetic db/db mice for 4 weeks did not result in significant differences in fasting blood glucose and Glycated Hemoglobin A1c (HbA1c) levels between MNAM-treated and untreated diabetic db/db mice. Additionally, there was no decrease in body weight observed in the MNAM-treated diabetic db/db mice; rather, there was a tendency for weight gain compared to the untreated diabetic db/db mice. It was also noted by Hong *et al.* (Hong et al., 2015) that MNAM supplementation did not prevent weight gain. The reason why MNAM is not as effective in controlling fasting glucose, HbA1c, and body weight *in vivo* can be explained by two factors. Firstly, MNAM is unstable *in vivo*. It can be quickly excreted through urine in the form of MNAM or MNAM oxides (Shibata et al., 1987; Shibata et al., 1988; Menon et al., 2007). Additionally, MNAM has a low permeability to the membrane, which is crucial for achieving the desired therapeutic effect of the drug (Neelakantan et al., 2018). These findings may partially explain the limited effectiveness of MNAM in controlling body weight and glucose homeostasis.

NNMTi is a NAM analog that inhibits NNMT activity by competing with NAM (Parsons and Facey, 2021). Evidence suggests that it can reverse HFD-induced obesity in mice (Neelakantan et al., 2018; Sampson et al., 2021). Neelakantan et al*.* (Neelakantan et al., 2018) observed that systemic administration of NNMTi to mice with diet-induced obesity markedly attenuated WAT mass and body weight, diminished adipocyte volume, and reduced plasma TC levels. NNMTi was shown to have high permeability to the membrane in a bidirectional permeation assay in Caco-2 cells. Importantly, NNMTi is highly selective and has no inhibitory effect on other SAM-dependent methyltransferases or NAD^+^ salvage pathway enzymes. *In vitro*, NNMTi significantly decreased the intracellular production of MNAM, increased the intracellular levels of NAD^+^, and inhibited adipogenesis. *In vitro*, mice treated with NNMTi exhibited a notable decrease in body weight, white fat mass, and adipocyte volume. Notably, NNMTi did not affect food intake or cause any observed side effects. The findings indicate that NNMTi is a promising inhibitor that targets NNMT and can reverse the obesity induced by diet and prevent the associated T2D. (Neelakantan et al., 2018). Similar to the NNMT knockdown experiments, NNMT inhibitors reversed obesity by modulating NAD^+^ rescue and the pathways mediated by SAM. These findings suggest that the combination of small molecule NNMT inhibitors with other nutritional supplements, such as the precursors of NAD^+^, may have the potential to enhance the efficacy of treatment for MetS and minimize the side effects caused by high pharmacological dosages (Roberti et al., 2021).

In addition to MNAM and NNMTi, three new NAM analog inhibitors were reported for the treatment of MetS-related disorders in animal models: **JBSNF-000088** (Kannt et al., 2018), **JBSNF-000265** (Ruf et al., 2018), and **JBSNF-000028** (Ruf et al., 2022). **JBSNF-000088** binds to the active site on NNMT and leads to the demethylation of SAM to SAH (Kannt et al., 2018). This inhibits NNMT-catalyzed methylation of NAM and reduces the production of MNAM. In animal models of metabolic diseases, **JBSNF-000028** (Ruf et al., 2022) and **JBSNF-000088** (Kannt et al., 2018) were identified to inhibit human and mouse NNMT activity. These compounds effectively reduced MNAM levels in adipose tissue, liver, and plasma, leading to improved insulin sensitivity and decreased body weight. Moreover, in a mouse model of diet-induced obesity and diabetes, they restored normal glucose tolerance. **JBSNF-000265** was discovered through Structure-Activity Relationship (SAR) studies (Ruf et al., 2018) as a compound that binds to NNMT in a manner similar to that of NAM. It has an improved ability to inhibit the enzymatic activity of NNMT and drastically reduces the formation of MNAM. When mice were orally administered 50 mg/kg of this compound, MNAM production decreased by approximately 80% within 2 h. Moreover, **RS004** (Horning et al., 2016) and 4-chloro-3-ethynylpyridine (Sen et al., 2019) were also reported to compete with NAM for binding by covalently linking to Cys165 and Cys159 in the active site of NNMT. However, their efficacy *in vivo* models has not been reported yet.

#### 4.2.3 Dual-substrate-competitive inhibitors

Dual-substrate-competitive inhibitors compete with the both substrates (SAM and NAM) for the binding sites in NNMT, thereby increasing inhibitors’ activity and selectivity (Gao et al., 2021). As shown in Table 1 and Figure 6, such inhibitors mainly include MvH45 (Van Haren et al., 2017), **AK-12** (Ruf et al., 2022), **MS2756** (Babault et al., 2018), **MS2734** (Babault et al., 2018), **GYZ-78** (Gao et al., 2019), **NS1** (Kuzmi et al., 2021), **LL320** (Chen D. et al., 2019), Yuanhuadine (Bach et al., 2018), and **CC-410** (Roberti et al., 2023). This class of inhibitors has been recently developed, thus there is a scarcity of reported cellular or *in vivo* data concerning these compounds. However, it will be intriguing to investigate whether this class of inhibitors targeting NNMT exhibits improved efficacy in this context. One notable dual-substrate NNMT inhibitor is Yuanhuadine, which is isolated from the flower buds of the traditional Chinese medicine Daphne genkwa. Some studies have reported that Yuanhuadine demonstrates a favorable inhibitory effect on the growth of various tumor cell lines (He, 2002; Zhang et al., 2006; Hong et al., 2011). Additionally, Roberti *et al.* (Roberti et al., 2023) reported a novel dual-substrate NNMT inhibitor, **CC-410**, which stably binds to and exhibits high specificity in inhibiting NNMT.

#### 4.2.4 Allosteric inhibitors

Allosteric inhibitors represent a class of inhibitors that do not compete for the SAM or NAM binding sites on NNMT. Instead, they bind to an allosteric site, thereby inhibiting NNMT activity. As previously mentioned, inhibitors targeting NNMT often bear structural resemblance to one or both of its substrates. As shown in Table 1 and Figure 6, in the pursuit of structurally diverse NNMT inhibitors, researchers have identified certain macrocyclic peptides that bind to NNMT, displaying potent inhibition with Half-maximal inhibitory concentration (IC_50_) values as low as 229 nM (Van Haren et al., 2021). Moreover, these macrocyclic peptides have been observed to downregulate MNAM production in cellular assays. Notably, these macrocyclic peptides exhibit noncompetitive behavior with either SAM or NAM, suggesting that they may function as allosteric inhibitors targeting NNMT (Van Haren et al., 2021).

## 5 Implications and recommendations for future research

As delineated in the text, NNMT is a crucial factor in MetS and has become a promising target for preventing or treating MetS-related diseases. However, despite the encouraging evidence, gaps in our knowledge remain to be filled in order to fully understand the mechanisms connecting NNMT with MetS-related disorders and to develop clinical agents against NNMT. The roles of NNMT in adipose tissues and livers appear to exhibit some contrasting effects. NNMT emerges as a novel regulator of adipose tissue function and energy expenditure. While NNMT influences energy metabolism in both adipose tissue and liver, its expression suppresses NAD^+^ levels specifically in adipose tissue. However, it is noteworthy that knockdown of NNMT does not seem to affect NAD^+^ levels in the liver, indicating potential divergent regulatory mechanisms of NNMT in these tissues and suggesting the existence of redundant pathways in NAD^+^ metabolism regulation (Kraus et al., 2014). Nonetheless, additional research is necessary to clarify the exact mechanisms behind NNMT’s actions in adipose tissues and liver. Specifically, it is necessary to determine how NNMT promotes fat accumulation in adipose tissues while managing fat accumulation in livers caused by nutrient overload (Trammell and Brenner, 2015). Further study also is needed to understand how NNMT inhibits the formation of NAD^+^ in adipose tissues and why there are redundant regulatory pathways for the metabolism of NAD^+^ in livers. This could provide insight into the functional differences of NNMT in different tissues.

Is NAD^+^ necessary for NNMT to regulate energy metabolism processes? The competition between NAD^+^ salvage and the methylation of NAM suggests that NNMT may reduce the oxidation of fuel and increase the storage of fat by regulating the formation of NAD^+^ (Trammell and Brenner, 2015). However, experiments have not demonstrated direct effects of NNMT on the intracellular levels of NAM and NAD^+^. In murine models, NNMT knockdown did not induce the accumulation of NAM in livers and adipose tissues, nor did it cause significant alterations in the intracellular levels of NAD^+^ in hepatocytes (Kraus et al., 2014; Hong et al., 2015). Therefore, it is possible that NNMT may regulate energy metabolism through other mechanisms, and that NAD^+^ is not necessary for this regulatory process.

Effects of NNMT on anaerobic energy metabolism remain unknown. Numerous studies have reported that NNMT has an inhibitory effect on the aerobic metabolism of sugars and fats, and NNMT Knockdown can increase oxygen consumption, promote sugar consumption and reduce fat synthesis, but the effects of NNMT on anaerobic energy metabolism have not been reported. Our previous study (Li et al., 2017) revealed higher expression levels of NNMT in the toe extensor muscle, typically reliant on anaerobic energy metabolism for power generation, compared to the soleus muscle, typically reliant on aerobic energy metabolism. Additionally, inhibition of NNMT using MNAM resulted in a decline in performance during anaerobic endurance exercise in rats (Zhou et al., 2018). These results suggest that NNMT may also contribute to the regulation of anaerobic energy metabolism.

Additional investigations are warranted to ascertain the suitability of NNMT as a therapeutic target for MetS. The development of RNAi drugs and small molecule inhibitors targeting NNMT is facing several challenges. For RNAi drugs, challenges include safety, efficacy, and delivery issues. To address these challenges, various delivery methods have been investigated, including viral vectors, non-viral vectors, and rational design strategies such as chemical modifications, cationic liposomes, polymers, nanocarriers, and biocoupled siRNAs, aimed at enhancing stability and intracellular delivery (Liu F. et al., 2018). Several RNAi drugs, including aptamers like pegaptanib targeting protein targets, and micro interfering RNAs like givosiran and patisiran, as well as antisense oligonucleotides like golodirsen and inotersen that interfere directly with RNA targets, have received FDA approval for medical use (Yu et al., 2020). Despite significant progress in clinical RNAi drug development, there remains considerable room for improvement in pharmacokinetics, pharmacodynamics, and strategies to mitigate toxicity (Setten et al., 2019). Regarding small molecule inhibitors targeting NNMT, challenges include the identification of efficient and selective inhibitors with robust *in vivo* activity (Barrows et al., 2022). Presently, NNMTi, a small molecule inhibitor of NNMT, has been widely employed to investigate the pharmacological effects of NNMT. However, the efficacy of small molecule inhibitors targeting NNMT remains uncertain due to their lack of selectivity and low metabolic stability (Iyamu and Huang, 2021).

## 6 Conclusion

NNMT is considered to play a significant role in MetS-related diseases. Inhibiting NNMT has been shown to increase energy expenditure, reduce fat accumulation, improve insulin sensitivity, and normalize glucose tolerance and fasting glucose levels. However, the precise mechanisms responsible for these effects are still unclear. Experiments utilizing various RNAi drugs and small molecule inhibitors targeting NNMT have highlighted its potential as a therapeutic target for preventing and treating diseases associated with MetS. Nevertheless, there have been no documented clinical trials focusing on NNMT to date. Further research is needed to elucidate the mechanisms by which NNMT operates in MetS and to develop therapeutic strategies targeting NNMT.
